# Anxiety matters: a pilot lab study into food, weight, and virtual body exposure in anorexia nervosa

**DOI:** 10.1186/s40337-024-01094-w

**Published:** 2024-09-13

**Authors:** Hanna Melles, Anita Jansen

**Affiliations:** Department of Clinical Psychological Science, Faculty of Psychology and Neuroscience, Maastricht University, P.O. Box 616, 6200 MD Maastricht, The Netherlands

**Keywords:** Anorexia nervosa, Anxiety, Food exposure, Weight exposure, Virtual body exposure, Exposure therapy

## Abstract

**Background:**

Anxiety is a core characteristic of anorexia nervosa and a potential target of exposure therapy, which requires a profound understanding of the patients’ fears in order to be successful. The knowledge about fears in anorexia nervosa that should be targeted during exposure therapy can be enriched by laboratory research to the precise emotional and behavioral responses of anorexia nervosa patients when they are exposed to disorder relevant fear stimuli.

**Methods:**

In the laboratory, patients with anorexia nervosa (n = 15) and healthy controls (n = 51) were exposed to 1. their own body weight and a 10% higher body weight on the scale, 2. a standardized lab breakfast, and 3. five virtual bodies with different BMIs ranging from extreme underweight to lower healthy weight. The participants emotional (anxiety, disgust, satisfaction, acceptance) and behavioral responses (calorie consumption) were assessed. Patients with anorexia nervosa but not the healthy controls then received an intensive exposure treatment (~ 30 individual exposure sessions) targeting their individual fears, next to standard care. After the exposure treatment, it was investigated whether the patients’ responses to the laboratory tasks changed.

**Results:**

Across all tasks, the patients reported more anxiety than healthy controls. The patients also consumed less calories during the breakfast and accepted the different body weights on the scale less than healthy controls. During the virtual body exposure, the patients’ emotional responses did not differ per avatar but they reacted more negatively towards avatars with healthier weights than did healthy controls. After the exposure treatment, the patients reported less fears and they consumed more calories while their BMIs had increased. They were also more accepting of healthier weights.

**Conclusions:**

Exposure to food-, body- and weight-related stimuli in the laboratory induces emotional reactions in patients with anorexia nervosa that are informative for the identification of exposure therapy treatment targets. In addition, exposure therapy targeting individual fears in patients with anorexia nervosa led to symptom reduction and is a promising intervention for the treatment of anorectic fears, though more research is needed to optimize its efficacy.

## Abstract (plain language summary)

Anxiety is an important characteristic of anorexia nervosa and a target of exposure therapy. Recent research revealed the variety of fears and other emotions (e.g. disgust) that patients with anorexia nervosa display, and that could be addressed in exposure therapy. More nuanced knowledge about potential treatment targets could be derived from experimental research which has so far mainly focused on the fear of food. We aimed to fill this gap by experimentally studying the behavioral and emotional responses of patients with anorexia nervosa (n = 15) and healthy controls (n = 51) when exposed to food-, body-, and weight-related stimuli in the laboratory, and then also tested whether the patients’ responses changed after they received an intensive exposure treatment. Patients were found to be more anxious about food and weight related stimuli, consumed fewer calories, and reacted more negatively when they imagined that virtual bodies of different weights were theirs, than did healthy controls. After the exposure treatment, patients reported less fears and they consumed more calories, while their BMIs had increased. They were also more accepting of healthier body weights. Exposure therapy is a promising intervention for the treatment of anorectic fears but more research is needed to optimize its effectiveness.

## Background

Anorexia nervosa is a severe eating disorder, characterized by a distorted body image, restrictive eating behaviours, a high illness burden and potentially serious physical consequences [1]. Anorexia nervosa has a poor prognosis and a mortality rate of ~5% makes it the most lethal mental disorder [2, 3]. High drop-out rates (between ~20–40%), non-response to treatments (~30%) and chronicity mark anorexia nervosa [4–7] and reflect how our current treatments are far from successful. Over the past decades, many interventions to treat anorexia nervosa were developed but it appears that none of these treatments consistently superiors another or outperforms control conditions [8, 9]. This leads to the suggestion that current treatments of anorexia nervosa do not yet target all important mechanisms of change.

A transdiagnostic factor that is involved in the development, maintenance of, and is predictive for the course and treatment outcomes of anorexia nervosa is anxiety [10–20]. Individuals with anorexia nervosa exhibit a variety of fears related to food, eating, weight gain and its social or personal consequences [21–25]. Patients, for instance, fear eating high-caloric foods alone or with others; they fear how the body might feel (e.g. full, uncomfortable, or sick) or how it might change after eating (e.g. weight gain). They also fear loss of control, negative judgement, and social rejection when they eat certain foods, gain weight or fail to meet their own standards of perfection [17, 21–24, 26]. It is assumed that learning processes including classical and operant conditioning are involved in the maintenance of such fears in anorexia nervosa [17, 20, 27, 28]. For instance, through classical conditioning, a stimulus such as food (CS; conditional stimulus) becomes associated with an aversive or feared outcome, such as immediate weight gain (US; unconditional stimulus) and by this food becomes a predictor of threat [20]. The mere presence of food is then sufficient to elicit fear and triggers fear reactions (CR; conditioned response). These fear reactions, called safety behaviours, include for instance food restriction, body checking, excessive exercise or purging [19, 20, 29]. By applying such behaviours, the patient can prevent the feared outcome and consecutively reduce fear in the present moment. Making immediate use of safety behaviours, however, also withholds the possibility to ascertain whether the initial fear is actually justified. Rather, through operant conditioning processes, the patient learns that safety behaviours are necessary to prevent the feared outcome and to reduce fear [19, 20]. So, in the long-term both the fear and safety behaviours will persist, or even increase [19, 30–32] and thereby possibly have severe consequences for the individuals’ psychological and physical health.

A promising intervention that aims at breaking such self-reinforcing circles of anxiety and safety behaviours is exposure therapy [30, 33, 34]. Its effectiveness is primarily known from the treatment of anxiety disorders, but an emerging line of research shows that it can also be relevant in the treatment of fears seen in anorexia nervosa (e.g. [16, 25, 35–43]. During exposure therapy, individuals with anorexia nervosa are repeatedly exposed to a feared stimulus (e.g. food) in the absence of the feared outcome (e.g. immediate weight-gain) and without the use of safety behaviors that purposefully prevent the occurrence of the feared outcome (e.g. excessive exercise after eating)[26, 44]. Recent research suggests that exposure therapy should be based on inhibitory learning principles, such as expectancy violation [30, 45]. This implies that during the exposure session, a clear hypothesis about the feared outcome is formulated, tested and violated, which supposedly leads to a decrease in the strength of believe in the expectancies and to a consecutive reduction of fear [26, 45–47].

Since a thorough understanding of the patients’ fears and safety behaviors is considered an inevitable pre-requisite to the success of exposure therapy [16, 26] more needs to be learned about fears in anorexia nervosa. So far, most knowledge on the presence and types of fears in anorexia nervosa either comes from intervention research or is based on self-report and questionnaire data. To date, only few experimental studies have examined anorectic fears in the laboratory (e.g. [48–50], although experimental research may be helpful in gathering more precise knowledge about the factors of maintenance and change that should be targeted in an effective eating disorder treatment [51, 52]. Next to knowing *that* patients with anorexia nervosa are afraid of certain food and body related stimuli, a better understanding of *how* and *when* exactly patients respond emotionally and behaviorally, or deviate from healthy controls when exposed to disorder relevant fear stimuli in the laboratory can be relevant in the optimization of treatments that target the fears, such as exposure therapy.

So far, previous laboratory studies on food related fears in anorexia nervosa have shown that as fear about eating increases, the maximum tolerated food portion size decreases in patients with anorexia nervosa [50] and that more pre-meal anxiety predicts less actual food intake [48]. This could particularly identify the fear experienced immediately before food consumption as an important point in time and relevant treatment target for exposure therapy [37, 39, 42]. Based on advances in research revealing the diversity of fears that play a role in eating disorders such as anorexia nervosa, it has been suggested that exposure treatments should target a broad set of fears and also other emotions, like disgust [36, 53]. In contrast to the study of fear of food, less is known about these other fears and emotions, like when in time they are most evident. Further, it is also not examined yet how emotions other than the fear of food can best be addressed during exposure therapy. While it is known that exposure to food, for instance, is the stimulus of interest when treating fears related to eating, less is known about other modalities such as scales or virtual reality applications to tackle eating disorder related emotions. Therefore, it is necessary to investigate both the type of emotional reactions that patients express, and the modality being used to stimulate such reactions in the laboratory. In addition, it is also relevant to study whether and which reactions to these stimuli will change after patients receive exposure therapy that targets their individual fears.

In the present study, we therefore exposed patients with anorexia nervosa and healthy controls to disorder relevant fear stimuli such as food and eating, weighing (exposure to their own and a 10% higher weight on the scale), and virtual bodies of various sizes (ranging from extreme underweight low normal weight) in the laboratory. We expected patients to consume less calories, to be more anxious during all exposures and to react more negatively towards higher weights on the scale and virtual bodies with rising weights, compared to healthy controls. To determine whether these cued fear responses are less strong after treatment, the patients completed the same laboratory exposure tasks again after they followed an intensive exposure intervention (~ 30 individual exposure sessions) following inhibitory learning principles, such as expectancy violation, next to standard care.

## Methods

### Participants

Female patients with anorexia nervosa and older than 16 years were recruited in two mental health centers in the Netherlands (Youz Maastricht; MUMC + Maastricht) and via flyers at Maastricht University. All patients with anorexia nervosa who were interested in participation were included, that is, we did not exclude participants for reasons of a very low or (partly) weight restored BMI, medication, comorbidities, or any other reasons. Initially n = 22 patients with anorexia nervosa were recruited. Of these, n = 7 dropped out and did not participate in the post measures, resulting in a final sample size of n = 15. At study begin, n = 9 (60%) patients reported being diagnosed with comorbid disorders (n = 5 depression, n = 2 OCD, n = 2 PTSD, n = 2 ADHD, n = 2 anxiety disorder, n = 1 skin picking disorder) and n = 5 (33.33%) were taking medication. Further, patients with anorexia nervosa had a lower BMI (M = 18.56, SD = 2.63; min = 14.12; max = 23.10) than the healthy controls. On average, patients with anorexia nervosa had an eating disorder for 4.93 years (SD = 3.88; min = 1; max = 12) and were in treatment for 3.27 years (SD = 3.33; min = 1; max = 10). N = 9 (60%) were following higher education (University). After completion of the study, each patient received a 70€ online voucher. The healthy control group consists of females of 16 years and older and was recruited via flyers and the research participation system of Maastricht University. Healthy controls were excluded when they were in psychological treatment currently or in the past three years, or when they reported to currently have a diagnosed mental disorder. This was assessed via a screening questionnaire at the beginning of the online survey. Participants in the healthy control group who scored high on the EDE-Q (global score > 2.5; [54] were also excluded. In the healthy control group, n = 77 healthy participants filled in the screening questionnaire and of these n = 16 did not meet the inclusion criteria. After screening, another n = 10 did not attend the lab measurement, leading to a final sample size of n = 51. The groups did not differ in age. After completion of the lab study, each healthy control participant received a 12.50€ online Amazon voucher or 1.5 study points. Demographic data and outcomes of the statistical analyses are reported in Table 1.

### Measures and procedure

#### Online questionnaires

*a) Demographic data:* Data on age, weight, height, nationality, education, diagnosis provided by their psychologist or psychiatrist, illness duration, and treatment information were collected.

For the sake of an elaborate sample description, the following questionnaires were used:

*b) Eating Disorder Examination Questionnaire (EDE-Q).* The EDE-Q 6.0 [55] assesses eating disorder psychopathology. The EDE-Q contains 28 items, each item is scored on a 0–6 scale, measuring the frequency or severity of eating disorder features over the past 28 days. Four subscales assessing *eating restraint*, *eating concern*, *shape concern* and *weight concern* are distinguished using 22 items, and their mean score reflects the global EDE-Q score. The remaining six items are open questions assessing compensatory behaviours, like binge-purge behaviours and excessive exercise. Higher scores mean more severe eating disorder psychopathology. Internal consistencies in the present study are Cronbach’s α = 0.87 for eating restraint, α = 0.87 for eating concern, α = 0.81 for weight concern, α = 0.92 for shape concern, and α = 0.95 for the global score.

*c) Fear of Food Measure (FOFM).* The FOFM [56] assesses fear of food. The self-report measure consists of 25 items that are rated from 1–7, and delivers three subscales: *anxiety about eating* (‘I feel tense when I am around food)’, *feared concern* which assesses maladaptive thoughts underlying eating anxiety (‘I worry that eating will make me dissatisfied with my body’) and *food avoidance behaviours* (‘There are certain foods I avoid because they make me anxious’). Item scores are summed to calculate the subscales and the total score. Higher scores mean more anxiety. Internal consistency of the subscales is Cronbach’s α = 0.97 for anxiety about eating, α = 0.93 for feared concern, α = 0.86 for food avoidance behaviours, and α = 0.97 for the total score.

*d) Eating Disorder Fear Questionnaire (EFQ).* The EFQ [24] assesses eating disorder related fears. The measure consists of 20 items, rated from 1–7 and delivers five subscales: *fear of weight gain* (‘I am afraid of gaining weight’), *fear of social consequences* (‘I am afraid that I will be judged if I gain weight’), *fear of personal consequences* (‘I fear that I will lose control of my life if I gain weight’), *fear of physical sensations* (‘I worry that I will not like how my body feels if I gain weight’) and *fear of social eating* (‘I am afraid of eating in public’). Item scores are averaged to calculate the subscales. Higher scores mean more anxiety. In the present study, internal consistency is Cronbach’s α = 0.96 for fear of weight gain, α = 0.94 for fear of social consequences, α = 0.87 for fear of personal consequences, α = 0.92 for fear of physical sensations, and α = 0.97 for fear of social eating.

*e) Depression Anxiety Stress Scale (DASS).* The anxiety subscale of the short 21-item version of the DASS [57] was used to assess general fear (‘I felt scared without any good reason’). Items are rated from 0–3. The subscale is calculated by summing the item scores and multiplying them by 2. Anxiety severity is categorized into normal (0–7), mild (8–9), moderate (10–14), severe (15–19), and extremely severe (20 +). Internal consistency in the present study is Cronbach’s α = 0.83.

*f) Intolerance of Uncertainty Scale (IUS).* The IUS [58] reflects how individuals react to ambiguous situations, implications of being uncertain as well as attempts to control the future (‘Uncertainty makes my life intolerable’). 27 items are rated from 1–5. Outcomes are summed to calculate a total score. Higher scores mean more intolerance of uncertainty. In the present study, internal consistency is Cronbach’s α = 0.94.

#### Lab exposure tasks

*a) Body weight exposure*. The task consisted of two phases in which the participant was instructed to stand on a scale and read aloud the weight presented on the screen. In the first phase, the scale displayed a body weight 10% above the actual body weight, and in the second phase the actual body weight was displayed. Each time before stepping on the scale, the participant was informed of what she would see: ‘The weight you will see is manipulated; it is 10% higher than your actual body weight’ or ‘your actual body weight will be presented on the screen’. Visual Analogue Scales (VASs; 0–100 mm) were used to assess anxiety, weight acceptance and weight satisfaction on a horizontal scale between 0 and 100. Anxiety was measured at baseline when entering the lab without seeing the scale, immediately before weighing, and after weighing, asking ‘How anxious are you now?’ (not at all anxious—extremely anxious). Acceptance of the displayed weight and satisfaction with the displayed weight were measured each time after the participant was weighed. Weight acceptance was assessed as ‘To what extent do you accept this weight at this moment?’ (not at all—completely) and weight satisfaction as ‘How satisfied are you with this weight at this moment?’ (very dissatisfied—very satisfied). All participants completed the task in the same order: + 10%, actual weight.

*b) Food exposure*. The participant was offered a standardized breakfast in the lab, consisting of vegetables, fruit, bread, various toppings, smoothies, coffee, and tea. The participant was then asked to imagine that she was on an adventure trip and that the food presented was the only meal she could eat that day. The task was to eat as much as the participant needed to get through the day. The time for the breakfast was set at 20 min and during this time, the experimenter left the room. Anxiety was measured at baseline when entering the lab without seeing the food, immediately before and after eating, with VASs asking ‘How anxious are you now?’ (not at all anxious—extremely anxious). The breakfast buffets were scheduled in the morning and participants were asked to not eat or drink anything, except for water and tea/coffee without sugar and milk, four hours before the appointment.

**Fig. 1:**
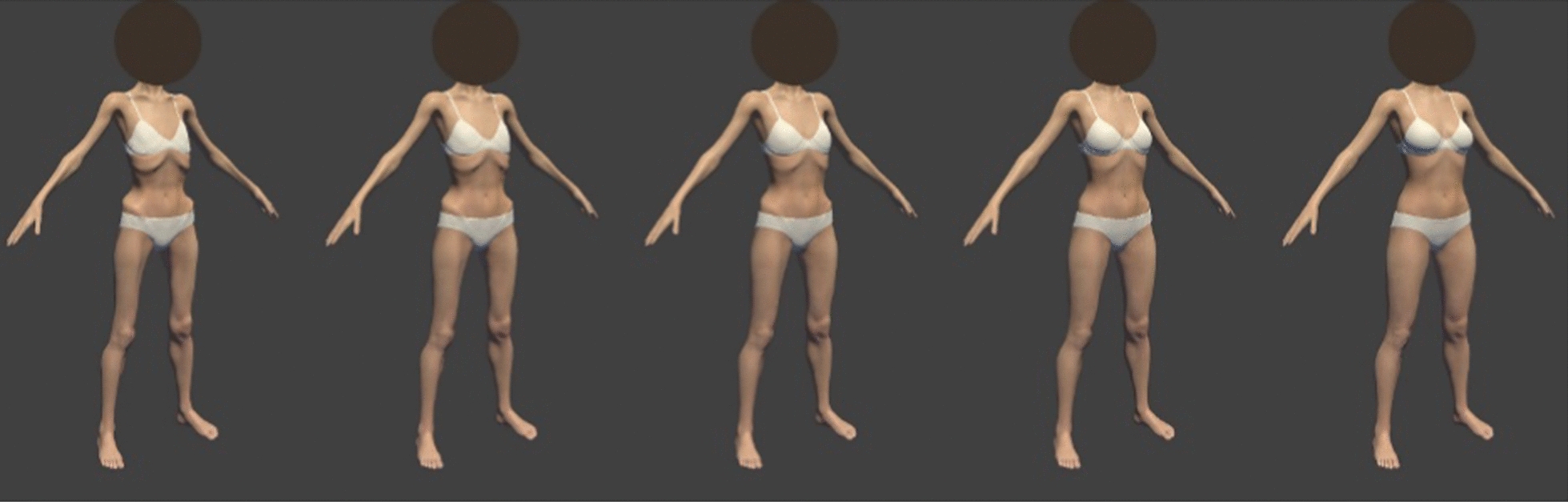
Avatars used during the virtual body exposure task. The five avatars used during the virtual body exposure task. From left to right they are coded as avatar 1–5. Avatar 1 represents an estimated BMI of approx. 14.5, avatar 2 approx. 15.5, avatar 3 approx. 16.5, avatar 4 approx. 17.5, and avatar 5 approx. 18.5

*c) Virtual body exposure*. In a virtual environment, five female avatars (see Fig. 1) standing in front of a virtual mirror were presented sequentially, for each participant in a new randomized order. The estimated BMI of the avatars varied from extreme underweight (BMI approx. 14.5), severe underweight (BMI approx. 15.5), moderate underweight (BMI approx. 16.5), mild underweight (BMI approx. 17.5) to low healthy weight (BMI approx. 18.5; see Fig. 1). In this paper, the avatars are referred to as avatar 1–5, with avatar 1 representing the severely underweight body and avatar 5 representing the low healthy weight body. Before the avatars were shown, the participant was instructed to imagine the avatar as her own body. In the virtual space, the participant was able to move the avatars’ arms and upper body by moving their own arms and upper body, which made it feel like the body was hers. Every avatar was presented for 30s, followed by virtual VASs assessing levels of body imagination (i.e., manipulation check), anxiety, body disgust, body acceptance and body satisfaction. Body imagination was measured asking ‘At this moment, how well can you imagine that this is your own body?’ (not good at all—very good), anxiety was measured asking ‘How anxious are you now?’ (not at all anxious—extremely anxious), body disgust as ‘How much disgust do you feel when looking at this body?’ (no disgust at all—very strong disgust), body acceptance as ‘To what extent do you accept this body at this moment?’ (not at all—completely), and body satisfaction as ‘How satisfied are you with this body at this moment?’ (very dissatisfied—very satisfied). Before a next avatar was presented, the participant was distracted in a virtual garden/playground for 90s.

### Procedure

**Fig. 2:**
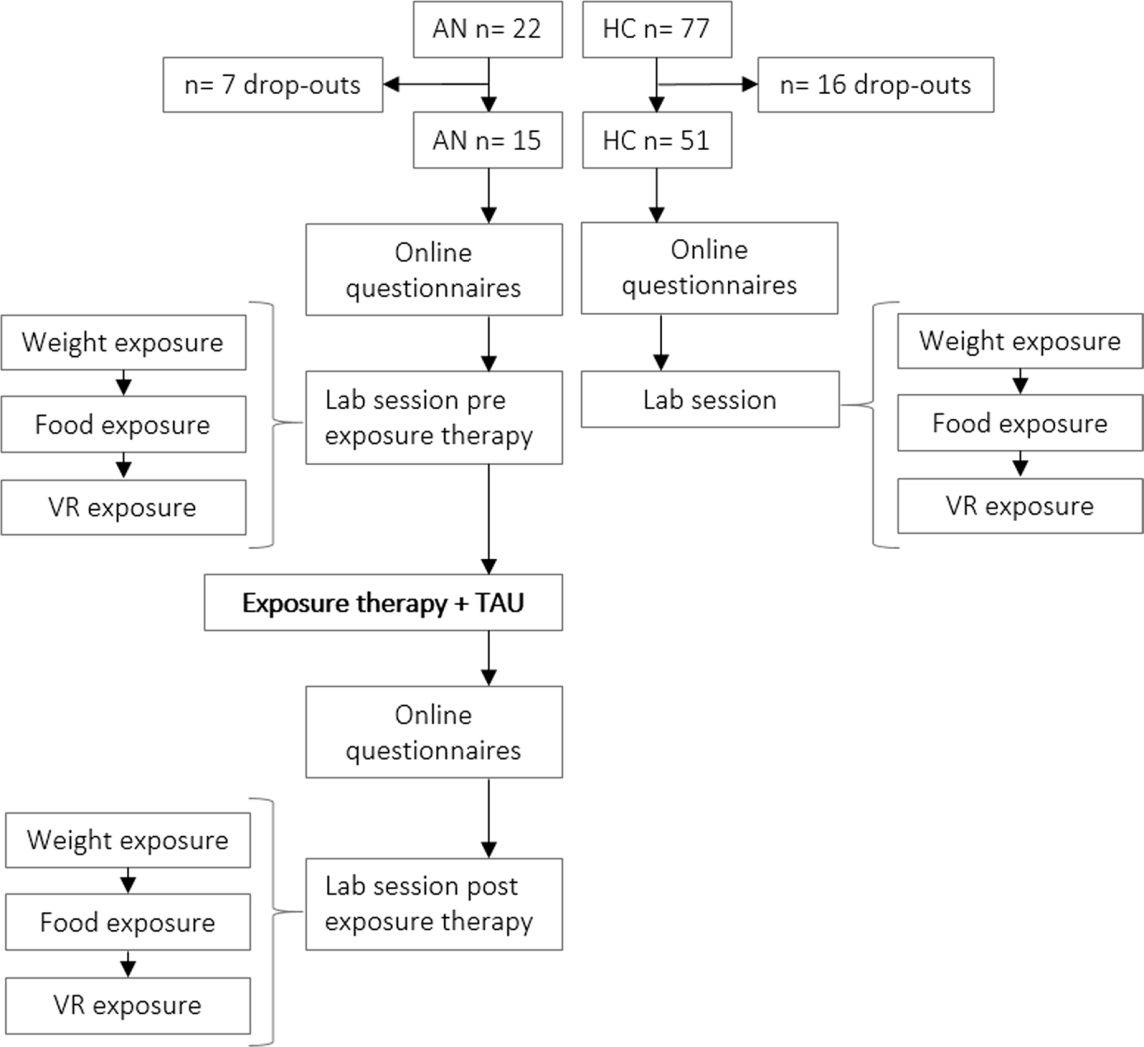
Procedure

After informed consent was obtained, participants completed the questionnaires online in the following order: screening questionnaire (only HC), demographics; Intolerance of Uncertainty Scale (IUS); Eating Disorder Fear Questionnaire (EFQ); Fear of Food Measure (FOFM); Depression Anxiety Stress Scale (DASS) and the Eating Disorder Examination Questionnaire (EDE-Q) which took approx. 45 min in total. About one week after completing the online questionnaires, the participant visited the lab and performed the exposure tasks in the following order: body weight exposure, food exposure, virtual body exposure. In addition, patients with anorexia nervosa then received an intensive exposure treatment (~ 30 exposure sessions per patient) following inhibitory learning principles, such as expectancy violation, delivered by two clinicians (H.M. and S.D) either together or alternating and next to the participants’ standard care (mostly CBT). The exposure sessions were scheduled two to three times per week and were tailored to the patients’ individual fears, threat outcomes and safety behaviours. Prior to each exposure exercise, therapist and patient formulated a concrete hypothesis about what the patient expected to happen (e.g. ‘when I eat a slice of pizza, I will gain 2kg immediately’). The hypothesis was then tested (exposure): in this case the patient was weighed, consumed the slice of pizza, and was weighed again afterwards. It was then discussed if the feared outcome occurred and what could be learned from the actual outcome (e.g. ‘when I eat a slice of pizza, I do not gain 2kg immediately’). The types of the exposure varied in content, time, context, and difficulty, depending on the patients’ specific threat expectancy. Common types of exposure included food/eating exposures, scale and weight exposures, mirror exposures, exposures to the tolerability of emotions related to food and weight gain, as well as virtual reality exposures (e.g. virtual eating alone or with others). See [26] for a more detailed description of the treatment rationale, procedure and practical examples of exposure sessions. After the intervention, the patients completed the same online questionnaires and lab tasks again. The study was approved by the Medical Ethics Review Committee (METC) of Maastricht UMC+ and the Ethical Committee of the Faculty of Psychology and Neuroscience, Maastricht University, the Netherlands. The procedure of the study is visualized in Fig. 2.

### Statistical analysis

Analyses were performed using JASP (version 0.17.1) and SPSS (version 27). For all tasks, acceptance and satisfaction scores correlated strongly (all r > 0.80). Therefore, acceptance and satisfaction variables were averaged into acceptance variables. The sociodemographic data are described with means and standard deviations (SD). Group differences (AN pre-therapy vs. healthy controls) on the demographic data and questionnaire outcomes were assessed with Welsh-t tests, effect size Cohen’s d. Due to the violation of assumptions, non-parametric analyses were used. As a non-parametric alternative to split-plot ANOVA, the within subjects effects were analyzed using Friedmann tests (effect size Kendall’s W). This was done for the various repeated measures variables (anxiety, acceptance, imagination, body disgust) across the tasks (food-, weight- and virtual body-exposure). The Friedmann tests were conducted separately for the AN ratings pre-therapy, AN ratings post-therapy and for the healthy controls. The between subjects differences (AN pre-therapy vs. healthy controls) on these repeated measures variables were then assessed with Mann–Whitney U tests. Bonferroni correction for multiple testing was used for post-hoc pairwise comparisons. Changes between pre- and post-treatment (AN pre-therapy vs. AN post-therapy) were examined using Wilcoxon signed-rank tests, effect size matched rank biserial correlation (rrb).

## Results

### Eating disorder psychopathology, eating disorder specific and general fears

With respect to eating disorder psychopathology, as expected, patients with anorexia nervosa scored higher than healthy controls on all subscales of the EDE-Q. After treatment, patients’ eating disorder psychopathology significantly reduced on the global score and the restraint and weight concern subscales but not on shape concern and eating concern. Concerning eating disorder specific and general fears, patients with anorexia nervosa scored higher than healthy controls on all subscales of the FOFM and EFQ as well as on the IUS and DASS anxiety subscale. After treatment, patients scored lower on all FOFM subscales (anxiety about eating, feared concerns, food avoidance behaviours) and on four out of five EFQ subscales (fear of weight gain, fear of social consequences, fear of physical sensations, fear of social eating) than before the exposure treatment. Means, standard deviations and outcomes of the statistical analyses are reported in Table 1.

**Table 1:** Eating disorder psychopathology, eating disorder specific and general fears

		AN pre therapy	AN post therapy	HC	AN pre therapy vs. HC	AN pre therapy vs. AN post therapy
		M (SD)	M (SD)	M (SD)		
Age		20.67 (2.06)		21.65 (3.91)	t(64) = − .931, *p* = .35	
BMI		18.56 (2.63)	19.47 (2.63)	21.97 (2.59)	t(22.61) = − 4.44, ***p* < .001**, d = − 1.31	z = − 2.84, ***p* = .003**, rrb = − .83
EDE-Q						
	Eating restraint	2.97 (1.51)	1.54 (1.45)	0.66 (0.74)	t(16.06) = 5.75, ***p* < .001**, d = 1.95	z = 2.55, ***p* = 0.01**, rrb = .80
	Eating concern	2.64 (1.49)	1.89 (1.32)	0.45 (0.61)	t(15.40) = 5.55, ***p* < .001**, d = 1.92	(*p* = .14)
	Weight concern	3.59 (1.43)	2.61 (1.27)	1.38 (1.07)	t(18.92) = 5.55, ***p* < .001**, d = 1.75	z = 2.89, ***p* < .01**, rrb = .88
	Shape concern	4.08 (1.13)	3.22 (1.59)	1.54 (1.11)	t(22.48) = 7.67, ***p* < .001**, d = 2.27	(*p* = .09)
	Global score	3.32 (1.10)	2.32 (1.30)	1.01 (0.72)	t(17.68) = 7.68, ***p* < .001**, d = 2.49	z = 2.60, ***p* < .01**, rrb = .79
FOFM	Anxiety about eating	36.13 (12.66)	25.07 (12.12)	11.35 (6.42)	t(16.17) = 7.31, ***p* < .001**, d = 2.47	z = 2.86, ***p* < .01**, rrb = .90
	Feared concerns	41.07 (10.85)	31.0 (12.94)	15.78 (8.63)	t(19.51) = 8.29, ***p* < .001**, d = 2.58	z = 2.76, ***p* < .01**, rrb = 8.84
	Food avoidance behaviors	29.87 (6.68)	20.64 (9.05)	12.98 (6.63)	t(22.75) = 8.61, ***p* < .001**, d = 2.53	z = 2.79, ***p* < .01**, rrb = .85
	Total score	107.07 (25.88)	76.71 (30.64)	40.12 (19.52)	t(18.93) = 9.27, ***p* < .001**, d = 2.92	z = 2.76, ***p* < .01**, rrb = .84
EFQ	Fear of weight gain	6.43 (0.59)	5.29 (1.63)	3.94 (1.66)	t(61.40) = 8.96, ***p* < .001**, d = 2.0	z = 2.04, ***p* = .04**, rrb = .67
	Fear of social consequences	5.02 (1.37)	4.46 (1.41)	3.13 (1.51)	t(24.80) = 4.59, ***p* < .001**, d = 1.31	z = 1.99, ***p* = .05**, rrb = .63
	Fear of personal consequences	5.17 (1.23)	4.69 (1.66)	2.62 (1.42)	t(25.89) = 6.8, ***p* < .001**, d = 1.92	*p* = .25
	Fear of physical sensations	6.64 (0.57)	5.67 (1.82)	4.57 (1.84)	t(63.70) = 6.99, ***p* < .001**, d = 1.52	z = 2.03, ***p* = .05**, rrb = .81
	Fear of social eating	4.23 (1.95)	2.36 (1.46)	1.58 (1.04)	t(16.38) = 5.06, ***p* < .001**, d = 1.7	z = 2.93, ***p* < .01**, rrb = .92
IUS		82.27 (19.81)	76.0 (21.44)	62.12 (16.50)	t(20.06) = 3.59, ***p* < .01**, d = 1.10	*p* = .27
DASS anxiety		14.93 (10.44)	13.14 (10.80)	6.08 (6.31)	t(17.11) = 3.12, ***p* < .01**, d = 1.03	*p* = .61

AN = anorexia nervosa; HC = healthy controls; age; BMI; EDE-Q = Eating Disorder Examination Questionnaire; FOFM = Fear of Food Measure; EFQ = Eating Disorder Fear Questionnaire; IUS = Intolerance of Uncertainty Scale; DASS = Depression, Anxiety, Stress Scale. The table reports means, standard deviations, comparisons between the groups (AN pre therapy vs. HC) and changes after the treatment (AN pre therapy vs. AN post therapy)

### Body weight exposure

#### Anxiety before and after exposure to different body weights

**Fig. 3:**
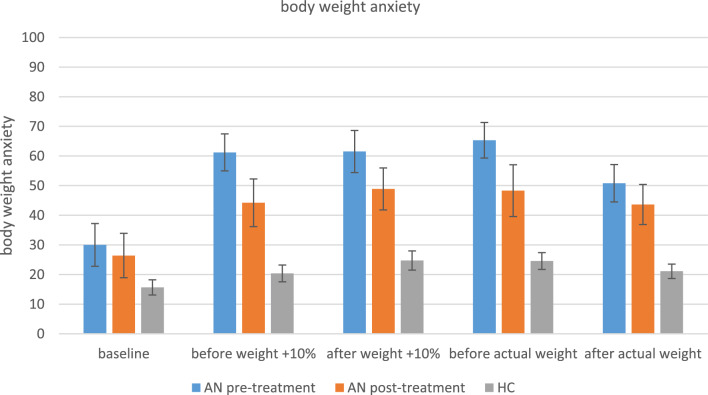
Anxiety before and after body weight exposure. AN = anorexia nervosa; HC = healthy controls. Anxiety levels at baseline (1), before (2) and after weight exposure (3) per condition (weight + 10% vs. actual weight), compared between groups (AN vs. HC) and pre- vs. post treatment (AN pre therapy vs. AN post therapy). Anxiety is measured between 0–100 where 0 means ‘no anxiety’ and 100 means ‘extreme anxiety’. *Means and standard deviations (SD)*: 1_preAN: 29.97 (27.97), 2_ + 10_preAN: 61.21 (25.67), 3_ + 10_preAN: 61.5 (27.42), 2_actual_preAN: 65.3 (23.28), 3_actual_preAN: 50.8 (24.41), 1_postAN: 26.4 (28.92), 2_ + 10_postAN: 44.21 (30.15), 3_ + 10_postAN: 48.89 (26.48), 2_actual_postAN: 48.29 (32.62), 3_actual_postAN: 43.63 (26.19), 1_HC: 15.66 (18.44), 2_ + 10%_HC: 20.3 (20.1), 3_ + 10%_HC: 24.73 (22.77), 2_actual_HC: 24.59 (20.21), 3_actual_HC: 21.1 (17.39)

Prior to the exposure treatment, the anxiety of patients with AN significantly increased after being informed that they would be exposed to a 10% higher body weight on the scale and anxiety did not decrease after body weight exposure. After being informed that they would now be exposed to their actual body weight, anxiety scores did not change and remained high after the exposure was finished. In contrast, the anxiety of healthy controls did not change throughout the entire body weight exposure task and they reported less anxiety than patients with AN at all time points. After the exposure treatment, patients with AN were less anxious before being exposed to a manipulated higher body weight on the scale than before treatment. Outcomes of the statistical analyses of the anxiety ratings are reported in Table 2 and shown in Fig. 3.

**Table 2:** Within subjects effects, between subjects effects and treatment outcomes of body weight anxiety

Anxiety	Main effect	Baseline vs. before weight + 10% exposure	Before weight + 10% vs. after weight + 10% exposure	After weight + 10% vs. before actual weight exposure	Before actual weight vs. after actual weight exposure
AN pre therapy	x^2^(4) = 31.66, ***p* < .001**, W = .53	t = 3.96, ***p***_**bonf**_** = .002**	t = .23, *p*_bonf_ = 1.0	t = .75, *p*_bonf_ = 1.0	t = 2.53, *p*_bonf_ = .144
AN post therapy	x^2^(4) = 14.88, ***p* = .005**, W = .27	t = 3.03, ***p***_**bonf**_** = .038**	t = .18, *p*_bonf_ = 1.0	t = .42, *p*_bonf_ = 1.0	t = .53, *p*_bonf_ = 1.0
HC	x^2^(4) = 31.77, *p* < .001, W = .16	t = 2.18, *p*_bonf_ = .306	t = 1.95, *p*_bonf_ = .526	t = .81, *p*_bonf_ = 1.0	t = .91, *p*_bonf_ = 1.0

Anxiety	Baseline	Before weight + 10% exposure	After weight + 10% exposure	Before actual weight exposure	After actual weight exposure
AN pre therapy vs. AN post therapy	z = .97, *p* = .359, rrb = .28	z = 1.98, ***p* = .049**, rrb = .60	z = 1.47, *p* = .153, rrb = .45	z = 1.51, *p* = .14, rrb = .46	z = .91, *p* = .389, rrb = .27
AN pre therapy vs. HC	z = − 2.04, ***p* = .041**	z = − 4.55, ***p* < .001**	z = − 4.05, ***p* < .001**	z = − 4.70, ***p* < .001**	z = − 3.99, ***p* < .001**

AN = anorexia nervosa; HC = healthy controls. The table shows anxiety measures before and after exposure to a manipulated higher weight or actual weight within the groups (AN pre therapy, AN post therapy, HC), comparisons between the groups (AN pre therapy vs. HC), and before vs. after the treatment (AN pre therapy vs. AN post therapy)

#### Weight acceptance after body weight exposure

**Fig. 4:**
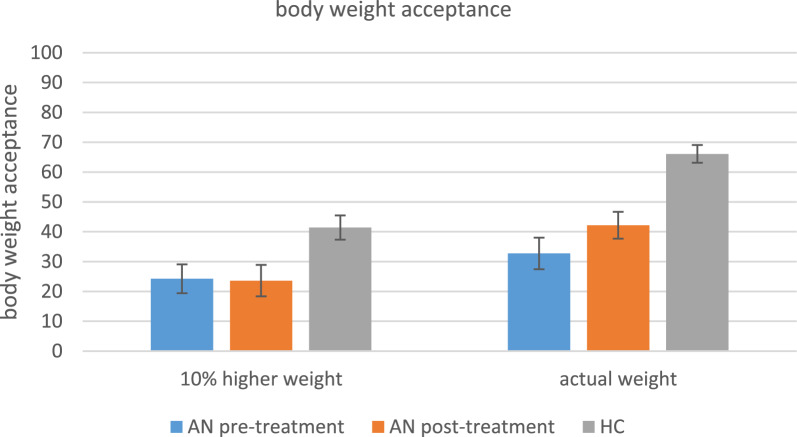
Body weight acceptance after body weight exposure. AN = anorexia nervosa; HC = healthy controls. Levels of body weight acceptance per condition (+ 10% vs. actual weight) compared between groups (AN vs. HC) and pre- vs. post-treatment (AN pre treatment vs. AN post treatment). Acceptance is measured between 0–100 where 0 means ‘no acceptance’ and 100 means ‘complete acceptance’. *Means and standard deviations (SD)*: + 10_preAN: 24.25 (18.81), + 10_postAN: 23.62 (19.72), actual_preAN: 32.75 (20.54), actual_postAN: 42.18 (17.38), + 10%_HC: 41.42 (28.45), actual_HC: 66.10 (21.30)

Patients with AN and healthy controls accepted their actual body weight more than a 10% higher body weight, but patients gave lower acceptance ratings than healthy controls for both body weights. After treatment, patients with anorexia nervosa accepted their actual weight marginally significant more than before treatment even though their BMIs had significantly increased. Outcomes of the statistical analyses of the acceptance ratings are reported in Table 3 and shown in Fig. 4.

To sum up, patients with anorexia nervosa were more anxious before and after body weight exposure and less accepting of the presented body weights than healthy controls. After treatment, the patients’ anxiety before exposure to a manipulated higher body weight decreased and acceptance of the actual body weight increased, even though they gained weight during the treatment.

**Table 3:** Within subjects effects, between subjects effects and treatment outcomes of body weight acceptance

	Weight + 10% vs. actual weight		Weight + 10%	Actual weight
AN pre therapy	z = − 2.44, ***p* = .012**, rrb = − .72	AN pre vs. AN post therapy	z = − .41, *p* = .715, rrb = − .12	z = − 1.87, ***p* = .065**, rrb = − .55
AN post therapy	z = − 2.54, ***p* = .012**, rrb = − .77	AN pre therapy vs. HC	z = − 2.08, ***p* = .037**	z = − 4.31, ***p* < .001**
HC	z = − 5.25, ***p* < .001**, rrb = − .86			

AN = anorexia nervosa; HC = healthy controls. The table shows acceptance ratings after exposure to a manipulated higher weight or actual weight within the groups (AN pre therapy, AN post therapy, HC), comparisons between the groups (AN pre therapy vs. HC), and before vs. after the treatment (AN pre therapy vs. AN post therapy)

### Food exposure

#### Food anxiety and calorie intake

**Fig. 5:**
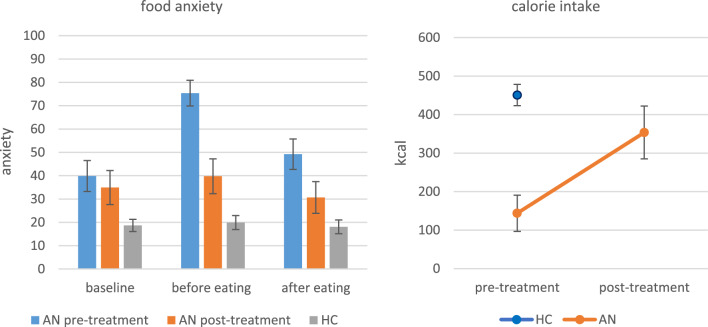
Food anxiety and calorie intake. AN = anorexia nervosa; HC = healthy controls. Left: Anxiety levels at baseline (1), before food exposure (2) and after food exposure (3); compared between groups (AN vs. HC) and pre- vs. post treatment (AN pre therapy vs. AN post therapy). Anxiety is measured between 0–100 where 0 means ‘no anxiety’ and 100 means ‘extreme anxiety’. Right: calorie intake (kcal) pre- and post-treatment (AN pre therapy vs. AN post therapy) and compared between groups (AN vs. HC). *Means and standard deviations (SD)*: 1_preAN: 39.87 (25.67), 2_preAN: 75.38 (21.29), 3_preAN: 49.2 (25.26), 1_postAN: 34.93 (28.26), 2_postAN: 39.75 (28.91), 3_postAN: 30.67 (26.41), 1_HC: 18.69 (18.53), 2_HC: 19.93 (21.33), 3_HC: 18.69 (21.09). Calories in kcal: pre_AN: 143.68 (182.38), post_AN: 353.49 (265.63), HC: 450.65 (196.90)

Before treatment: when patients were exposed to the breakfast, their anxiety increased significantly. After the food exposure, anxiety significantly reduced to a level that did not differ from the baseline anxiety reported at the beginning of the food exposure task. Anxiety of the healthy controls did not change during food exposure and they were less anxious than the patients at all time points. After treatment: pre-meal anxiety and anxiety after eating were significantly lower than before treatment. The patients generally ate less than the healthy controls, but they consumed more than twice as many calories after treatment compared to before, while their BMI had increased. Outcomes of the statistical analyses of the anxiety ratings are reported in Table 4 and shown in Fig. 5.

In sum, patients were more anxious when exposed to food and they consumed less calories than healthy controls. After treatment the patients’ anxiety was reduced and caloric intake increased despite weight gain.

**Table 4:** Within subjects effects, between subjects effects and treatment outcomes of food anxiety

Anxiety	Main effect	Baseline vs. before food exposure	Before food vs. after food exposure	Baseline vs. after food exposure
AN pre therapy	x^2^(2) = 13.66, ***p* = .001**, W = .45	t = 3.41, ***p***_**bonf**_** = .006**	t = 2.95, ***p***_**bonf**_** = .019**	t = .46, *p*_bonf_ = 1.0
AN post therapy	x^2^(2) = 8.78, ***p* = .012**, W = .29	t = 2.12, *p*_bonf_ = .129	t = 2.86, ***p***_**bonf**_** = .024**	t = .74, *p*_bonf_ = 1.0
HC	x^2^(2) = 1.38, *p* = .502, W = .01	t = .15, *p*_bonf_ = 1.0	t = .93, *p*_bonf_ = 1.0	t = 1.09, *p*_bonf_ = .84

Anxiety	Baseline	Before food exposure	After food exposure	kcal
AN pre therapy vs. AN post therapy	z = .79, *p* = .454, rrb = .23	z = 3.12, ***p* = .002,** rrb = .92	z = 2.04, ***p* = .041**, rrb = .60	z = − 3.30, ***p* = .001**, rbb = − 1.0
AN pre therapy vs. HC	z = − 3.26, ***p* = .001**	z = − 5.35, ***p* < .001**	z = − 4.07, ***p* < .001**	t = − 5.62, ***p* < .001**, d = − 1.62

AN = anorexia nervosa; HC = healthy controls. The table shows anxiety measures before and after exposure to food within the groups (AN pre therapy, AN post therapy, HC), comparisons between the groups (AN pre therapy vs. HC), and before vs. after the treatment (AN pre therapy vs. AN post therapy)

### Virtual body exposure

#### Manipulation check: body imagination

**Fig. 6:**
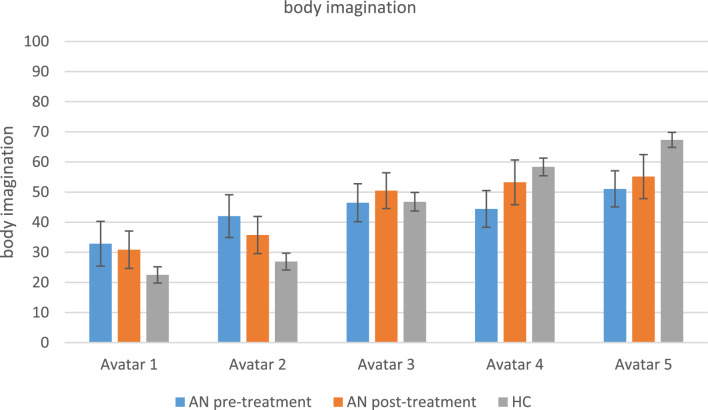
Body imagination. AN = anorexia nervosa; HC = healthy controls. Levels of body imagination per avatar (1–5); pre- and post-treatment (AN pre treatment vs. AN post treatment), compared between the groups (AN vs. HC). The variables are measured between 0–100 where 0 means that imagination was not good at all and 100 means that imagination was very good. *Means and standard deviations (SD)*: 1_preAN: 32.85 (28.85), 2_preAN: 42.02 (27.51), 3_preAN: 46.46 (24.42), 4_preAN: 44.41 (23.55), 5_preAN: 51.05 (23.22), 1_postAN: 30.88 (24.07), 2_postAN: 35.72 (23.90), 3_postAN: 50.47 (23.01), 4_postAN: 53.23 (28.77), 5_postAN: 55.13 (28.35), 1_HC: 22.51 (19.22), 2_HC: 26.91 (20.09), 3_HC: 46.78 (22.02), 4_HC: 58.35 (20.91), 5_HC: 67.31 (17.72)

After each virtual body exposure, the participants rated how well they could imagine that the avatar was their own body. The imagination ratings of anorexia nervosa patients did not differ between avatars and they could embody a very thin avatar (no. 2) better than healthy controls, whereas they could embody the more healthy weight bodies (no. 4 & 5) less than the healthy controls. Healthy controls could embody the avatars increasingly better, the higher their weight became. For both groups, the imagination ratings were rather moderate. Outcomes of the statistical analyses of the imagination ratings are reported in Tables 5 and 6 and are shown in Fig. 6.

#### Anxiety before virtual body exposure

**Fig. 7:**
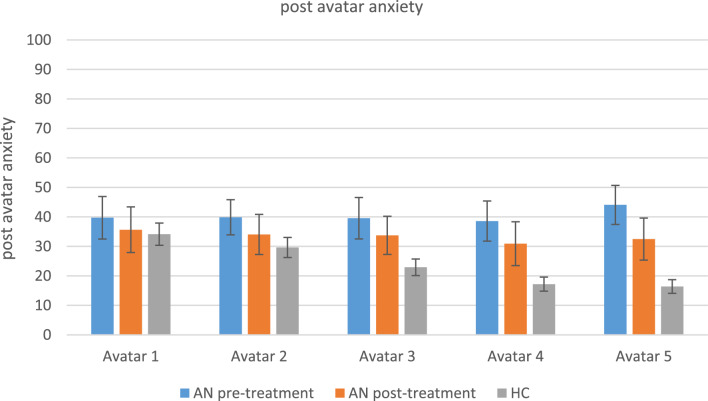
Anxiety after virtual body exposure. AN = anorexia nervosa; HC = healthy controls. Levels of anxiety per avatar (1–5) after virtual body exposure compared between groups (AN vs. HC) and pre- vs. post treatment (AN pre vs. AN post treatment). Anxiety is measured between 0–100 where 0 means ‘no anxiety’ and 100 means ‘extreme anxiety’. *Means and standard deviations (SD)* per time point: baseline (a), before virtual body exposure (b), and after virtual body exposure (c): a_preAN: 36.80 (30.64), 1_b_preAN: 37.70 (25.71), 2_ b_preAN: 32.72 (25.37), 3_ b_preAN: 38.50 (29.69), 4_ b_preAN: 35.98 (24.67), 5_ b_preAN: 32.90 (23.86), 1_c_preAN: 39.7 (27.93), 2_c_preAN: 39.86 (23.08), 3_c_preAN: 39.54 (27.28), 4_c_preAN: 38.57 (26.41), 5_c_preAN: 44.05 (25.69), a_postAN: 23.07 (21.73), 1_b_postAN: 24.21 (24.34), 2_b_postAN: 25.37 (22.72), 3_b_postAN: 29.79 (24.72), 4_b_postAN: 23.86 (21.51), 5_b_postAN: 24.93 (24.33), 1_c_postAN: 35.64 (30.02), 2_c_postAN: 34.03 (26.36), 3_c_postAN: 33.73 (25.22), 4_c_postAN: 30.91 (28.79), 5_c_postAN: 32.47 (27.60), a_HC: 23.69 (25.72), 1_b_HC: 17.01 (19.41), 2_b_HC: 18.04 (19.61), 3_b_HC: 18.36 (22.23), 4_b_HC: 18.46 (19.87), 5_b_HC: 18.04 (19.65), 1_c_HC: 34.13 (27.06), 2_c_HC: 29.63 (24.40), 3_c_HC: 22.90 (20.07), 4_c_HC: 17.19 (17.05), 5_c_ HC: 16.38 (16.66)

Prior to treatment, patients generally reported higher levels of anxiety before virtual body exposure than healthy controls, but for both groups anxiety did not differ between avatars. After treatment, patients were more afraid before exposure to the skinniest avatar (no. 1) than before treatment. Outcomes of the statistical analyses of the anxiety ratings are reported in Tables 5 and 6 and in Fig. 7.

#### Anxiety after virtual body exposure

Before treatment, patients’ anxiety ratings after virtual body exposure did not differ between avatars, and they were more anxious than healthy controls for the avatars that were not extremely skinny (no. 3, 4 and 5). Healthy controls were more anxious after exposure to skinny avatars compared to avatars with more healthy weights (no. 1 & 2 vs. 4 & 5; no. 3 vs. 5). The anxiety scores of the patients did not significantly change from pre- to post treatment. Outcomes of the statistical analyses of the anxiety ratings are reported in Tables 5 and 6 and are shown in Fig. 7.

#### Body disgust after virtual body exposure

**Fig. 8:**
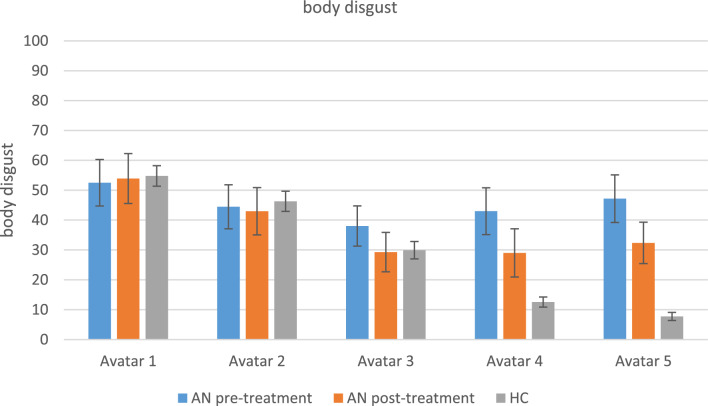
Body disgust after virtual body exposure. AN = anorexia nervosa; HC = healthy controls. Levels of body disgust per avatar (1–5), compared between groups (AN vs. HC) and pre- vs. post treatment (AN pre therapy vs. AN post therapy). The variables are measured between 0–100 where 0 means ‘no disgust’ and 100 means ‘very strong disgust’. *Means and standard deviations (SD)*: 1_preAN: 52.49 (30.18), 2_preAN: 44.44 (28.50), 3_preAN: 38.01 (26.07), 4_preAN: 42.98 (30.34), 5_preAN: 47.16 (30.83), 1_postAN: 53.91 (32.39), 2_postAN: 42.96 (30.68), 3_postAN: 29.27 (25.54), 4_postAN: 28.99 (31.27), 5_postAN: 32.35 (26.77), 1_HC: 54.77 (24.61), 2_HC: 46.28 (23.99), 3_HC: 29.91 (20.85), 4_HC: 12.55 (12.16), 5_HC: 7.74 (9.78)

Before treatment, the patients’ disgust ratings after virtual body exposure did not differ per avatar. The disgust ratings of the healthy controls did not differ for the very skinny avatars (no. 1 & 2) but gradually decreased as the avatars got healthier weights. The groups did not significantly differ in their disgust for the skinnier avatars (no. 1, 2 & 3) but patients with AN were significantly more disgusted by the more healthy weight avatars (no. 4 & 5) than healthy controls. After treatment, the patient’s disgust ratings did not significantly change. Outcomes of the statistical analyses of the disgust ratings are reported in Tables 5 and 6 and shown in Fig. 8.

#### Body acceptance after virtual body exposure

**Fig. 9:**
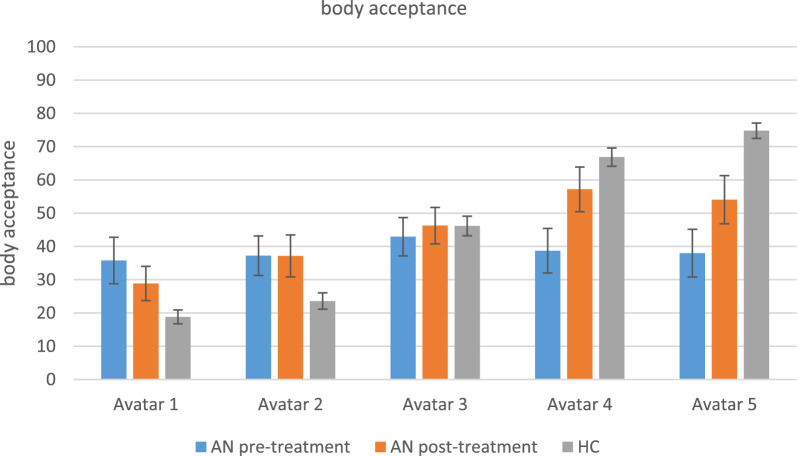
Body acceptance after virtual body exposure**.** AN = anorexia nervosa; HC = healthy controls. Levels of body acceptance per avatar (1–5); compared between groups (AN vs. HC) and pre- vs. post treatment (AN pre therapy vs. AN post therapy). Acceptance is measured between 0–100 where 0 means ‘no acceptance’ and 100 means ‘complete acceptance’. *Means and standard deviations (SD)*: 1_preAN: 35.76 (27.02), 2_preAN: 37.22 (22.95), 3_preAN: 42.91 (22.30), 4_preAN: 38.70 (25.95), 5_preAN: 37.98 (27.01), 1_postAN: 28.86 (20.0), 2_postAN: 37.16 (24.43), 3_postAN: 46.25 (21.24), 4_postAN: 57.16 (25.94), 5_postAN: 54.04 (28.03), 1_HC: 18.01 (14.84), 2_HC: 23.58 (17.54), 3_ HC: 46.13 (21.02), 4_HC: 66.85 (19.62), 5_HC: 74.78 (16.56)

Before treatment, acceptance ratings of AN patients did not differ between the avatars. Healthy controls did not differ in how much they accepted the very skinny avatars (no. 1 & 2) but their acceptance ratings steadily increased towards the avatars with more healthy weights. Further, patients with AN accepted the very skinny avatars (no. 1 & 2) more than healthy controls and the more healthy weight avatars (no. 4 & 5) less than healthy controls. After treatment, patients accepted the skinniest avatars (no. 1 & 2) less than a more normal weight avatar (no. 4) and they generally accepted this avatar (no. 4) marginally significant more after the treatment than before. Outcomes of the statistical analyses of the acceptance ratings are reported Tables 5 and 6 and depicted in Fig. 9.

To sum up, before treatment, the anxiety ratings, body disgust ratings and body acceptance ratings of patients with anorexia nervosa after virtual body exposure did not differ regardless of the avatar’s BMI. Patients were also more afraid, more disgusted and less accepting of avatars with healthier weights and more accepting of very skinny avatars than healthy controls. Healthy controls responded less anxious, less disgusted and more accepting with increasing body weights of the avatars. After treatment, patients were more accepting of a healthier weight avatar.

**Table 5:** Within subjects effects of the virtual reality measures

		Main effect	1 vs. 2	1 vs. 3	1 vs. 4	1 vs. 5	2 vs. 3	2 vs. 4	2 vs. 5	3 vs 4	3 vs. 5	4 vs. 5
Imagination	AN pre therapy	x^2^(4) = 7.09, *p* = .131, W = .12	t = 1.94, *p*_bonf_ = .58				t = .34, *p*_bonf_ = 1.0			t = .68, *p*_bonf_ = 1.0		t = .57, *p*_bonf_ = 1.0
	AN post therapy	x^2^(4) = 21.17, ***p* < .001**, W = .35	t = 1.02, *p*_bonf_ = 1.0		t = 3.42, ***p***_**bonf**_** = .012**	t = 3.76, ***p***_**bonf**_** = .004**	t = 1.59, *p*_bonf_ = 1.0			t = .80, *p*_bonf_ = 1.0		t = .34, *p*_bonf_ = 1.0
	HC	x^2^(4) = 131.84, ***p* < .001**, W = .65	t = 1.06, *p*_bonf_ = 1.0	t = 5.55, ***p***_**bonf**_** < .001**	t = 7.36, ***p***_**bonf**_** < .001**	t = 9.42, ***p***_**bonf**_** < .001**	t = 4.49, ***p***_**bonf**_** < .001**	t = 6.30, ***p***_**bonf**_** < .001**	t = 8.36, ***p***_**bonf**_** < .001**	t = 1.81, *p*_bonf_ = .72	t = 3.87, ***p***_**bonf**_** = .001**	t = 2.06, *p*_bonf_ = .41
Pre avatar anxiety	AN pre therapy	x^2^(4) = 3.15, *p* = .534, W = .05	t = 1.48, *p*_bonf_ = 1.0				t = .91, *p*_bonf_ = 1.0			t = .0, *p*_bonf_ = 1.0		t = .80, *p*_bonf_ = 1.0
	AN post therapy	x^2^(4) = 7.47, *p* = .113, W = .12	t = .68, *p*_bonf_ = 1.0				t = 1.82, *p*_bonf_ = .738			t = 1.59, *p*_bonf_ = 1.0		t = .45, *p*_bonf_ = 1.0
	HC	x^2^(4) = 4.0, *p* = .406, W = .02	t = .12, *p*_bonf_ = 1.0				t = .44, *p*_bonf_ = 1.0			t = 1.75, *p*_bonf_ = .82		t = .44, *p*_bonf_ = 1.0
Post avatar anxiety	AN pre therapy	x^2^(4) = 2.93, *p* = .569, W = .05	t = .68, *p*_bonf_ = 1.0				t = .34, *p*_bonf_ = 1.0			t = .45, *p*_bonf_ = 1.0		t = .80, *p*_bonf_ = 1.0
	AN post therapy	x^2^(4) = 2.51, *p* = .643, W = .04	t = 1.02, *p*_bonf_ = 1.0				t = .57, *p*_bonf_ = 1.0			t = .80, *p*_bonf_ = 1.0		t = 1.14, *p*_bonf_ = 1.0
	HC	x^2^(4) = 35.40, ***p* < .001**, W = .17	t = .81, *p*_bonf_ = 1.0		t = 4.24, ***p***_**bonf**_** < .001**	t = 4.68, ***p***_**bonf**_** < .001**	t = .69, *p*_bonf_ = 1.0	t = 3.43, ***p***_**bonf**_** = .007**	t = 3.87, ***p***_**bonf**_** = .001**	t = 2.74, *p*_bonf_ = .066	t = 3.18, ***p***_**bonf**_** = .017**	t = .44, *p*_bonf_ = 1.0
Body disgust	AN pre therapy	x^2^(4) = 4.16, *p* = .385, W = .07	t = 1.14, *p*_bonf_ = 1.0				t = .34, *p*_bonf_ = 1.0			t = .34, *p*_bonf_ = 1.0		t = 1.14, *p*_bonf_ = 1.0
	AN post therapy	x^2^(4) = 12.64, *p* = .013, W = .21	t = .11, *p*_bonf_ = 1.0				t = 2.39, *p*_bonf_ = .202			t = .0, *p*_bonf_ = 1.0		t = .80, *p*_bonf_ = 1.0
	HC	x^2^(4) = 143.31, ***p* < .001**, W = .70	t = 1.25, *p*_bonf_ = 1.0	t = 5.24, ***p***_**bonf**_** < .001**	t = 8.12, ***p***_**bonf**_** < .001**	t = 9.73, ***p***_**bonf**_** < .001**	t = 3.99, ***p***_**bonf**_** < .001**	t = 6.86, ***p***_**bonf**_** < .001**	t = 8.48, ***p***_**bonf**_** < .001**	t = 2.87, ***p***_**bonf**_** = .046**	t = 4.49, ***p***_**bonf**_** < .001**	t = 1.62, *p*_bonf_ = 1.0
Body acceptance	AN pre therapy	x^2^(4) = 1.01, *p* = .907, W = .02	t = .34, *p*_bonf_ = 1.0				t = .34, *p*_bonf_ = 1.0			t = .23, *p*_bonf_ = 1.0		t = .80, *p*_bonf_ = 1.0
	AN post therapy	x^2^(4) = 16.96, ***p* = .002**, W = .28	t = .0, *p*_bonf_ = 1.0		t = 3.19, ***p***_**bonf**_** = .023**		t = 2.39, *p*_bonf_ = .202	t = 3.19, ***p***_**bonf**_** = .023**		t = .80, *p*_bonf_ = 1.0		t = 1.37, *p*_bonf_ = 1.0
	HC	x^2^(4) = 159.07, ***p* < .001**, W = .78	t = 1.56, *p*_bonf_ = 1.0	t = 5.30, ***p***_**bonf**_** < .001**	t = 8.67, ***p***_**bonf**_** < .001**	t = 10.35, ***p***_**bonf**_** < .001**	t = 3.74, ***p***_**bonf**_** = .002**	t = 7.11, ***p***_**bonf**_** < .001**	t = 8.79, ***p***_**bonf**_** < .001**	t = 3.37, ***p***_**bonf**_** = .009**	t = 5.05, ***p***_**bonf**_** < .001**	t = 1.68, *p*_bonf_ = .937

AN = anorexia nervosa; HC = healthy controls. Within subjects outcomes are reported separately per group (AN pre therapy, AN post therapy, HC) for the various VR measures (imagination, pre avatar anxiety, post avatar anxiety, body disgust, body acceptance). The table shows comparisons between the different avatars (1-5) in rising order (1 vs. 2, 2 vs. 3, 3 vs. 4, 4 vs. 5) as well as significant findings of additional comparisons. The remaining comparisons not reported in the table were not significant

**Table 6:** Between subjects effects and treatment outcomes of the virtual reality measures

		Baseline	Avatar 1	Avatar 2	Avatar 3	Avatar 4	Avatar 5
Imagination	AN pre therapy vs. AN post therapy		z = .34, *p* = .754, rrb = .10	z = 1.22, *p* = .233, rrb = .37	z = − .28, *p* = .802, rrb = − .09	z = − .66, *p* = .53, rrb = − .20	z = − .72, *p* = .49, rrb = − .22
	AN pre therapy vs. HC		z = − 1.26, *p* = .207	z = − 1.97, ***p* = .049**	z = − .01, *p* = .994	z = − 1.95, ***p* = .051**	z = − 2.47, ***p* = .013**
Pre avatar anxiety	AN pre therapy vs. AN post therapy	z = 1.15, *p* = .263, rrb = .36	z = 2.04, ***p* = .045**, rrb = .62	z = 1.79, *p* = .08, rrb = .54	z = .47, *p* = .66, rrb = .14	z = 1.85, *p* = .069, rrb = .56	z = .91, *p* = .379, rrb = .28
	AN pre therapy vs. HC	z = − 1.97, ***p* = .049**	z = − 3.24, ***p* = .001**	z = − 2.12, ***p* = .034**	z = − 2.76, ***p* = .006**	z = − 2.46, ***p* = .014**	z = − 2.36, ***p* = .018**
Post avatar anxiety	AN pre therapy vs. AN post therapy		z = .72, *p* = .49, rrb = .22	z = 1.29, *p* = .201, rrb = .39	z = .78, *p* = .45, rrb = .24	z = 1.04, *p* = .315, rrb = .31	z = 1.66, *p* = .103, rrb = .50
	AN pre therapy vs. HC		z = − .70, *p* = .486	z = − 1.55, *p* = .120	z = − 2.20, ***p* = .028**	z = − 2.99, ***p* = .003**	z = − 3.76, ***p* < .001**
Body disgust	AN pre therapy vs. AN post therapy		z = − .22, *p* = .851, rrb = − .07	z = − .22, *p* = .851, rrb = .07	z = 1.16, *p* = .258, rrb = .35	z = 1.02, *p* = .330, rrb = .30	z = 1.54, *p* = .132, rrb = .47
	AN pre therapy vs. HC		z = − .24, *p* = .813	z = − .27, *p* = .789	z = − .94, *p* = .347	z = − 3.92, ***p* < .001**	z = − 4.98, ***p* < .001**
Body acceptance	AN pre therapy vs. AN post therapy		z = 1.22, *p* = .233, rrb = .37	z = .41, *p* = .706, rrb = .12	z = .09, *p* = .95, rrb = .03	z = 1.98, ***p* = .052,** rrb = − .60	z = − 1.54, *p* = .13, rrb = − .47
	AN pre therapy vs. HC		z = − 2.26, ***p* = .024**	z = − 2.20, ***p* = .028**	z = − .24, *p* = .813	z = − 3.50, ***p* < .001**	z = − 4.43, ***p* < .001**

AN = anorexia nervosa; HC = healthy controls. The table reports group differences (AN pre therapy vs. HC) among the various VR measures (imagination, pre avatar anxiety, post avatar anxiety, body disgust, body acceptance) and treatment effects on those measures (AN pre therapy vs. AN post therapy)

## Discussion

In the present study, the emotional and behavioral responses of patients with anorexia nervosa and healthy controls when exposed to food, eating, weighing, and virtual bodies were examined in the laboratory, and it was assessed if the reactions of anorexia nervosa patients to these lab measures changed after they received an intensive exposure treatment using inhibitory leaning principles, next to standard care. Our hypotheses were partly confirmed: Patients reported more anxiety during food and weight exposure, consumed less calories, and accepted the body weights they were exposed to less than healthy controls. Patients were also more afraid, more disgusted, and less accepting of more healthier-weight virtual bodies, while they accepted extremely skinny bodies more than the healthy controls. As predicted, healthy controls reacted less anxious, less disgusted and more accepting with rising weights of the virtual bodies. Interestingly, the patients’ reactions did not differ regardless of the displayed weight on the scale or the avatars’ body weight. After exposure treatment, patients reported less fears on the questionnaires, and they were less anxious before exposure to food and a manipulated body weight on the scale, while their BMIs had significantly increased, they consumed more calories, and were more accepting of healthier weights.

Finding that patients with anorexia nervosa were more anxious when exposed to different body weights on the scale and accepted the presented weights less than healthy controls supports the general notion that fears related to body weight are key characteristics of anorexia nervosa [1]. Similarly, patients with anorexia nervosa displayed more pre- and post-meal anxiety, and consumed less calories than healthy controls when exposed to food, which is consistent with previous research [48, 49]. It is interesting, that fear levels of patients were about equally high before and after both weight exposures and for both presented weights (actual body weight and 10% increased body weight), and that pre-meal anxiety was so high although food consumption itself was voluntary and could therefore even be avoided, if wanted. Detecting that the patients’ fear was high before the breakfast began and before being weighed may align with the fear-learning model outlined in the introduction, according to which the mere presence of the CS is sufficient to activate fear memories and to trigger fear reactions. Accordingly, in this study, the confrontation with food cues or food related situations seemed to trigger fear even before any food was consumed; similarly, exposure to the scale itself appeared to elicit fear, before the patient was weighed or confronted with specific weights on the screen. Showing that the anticipation of, for example, being weighed and eating activates severe fears in patients with anorexia nervosa but not in healthy controls may be useful for identifying treatment targets for exposure therapy. This requires a profound understanding of the patients’ fears to be effective [16]. Finding that anxiety is highest before the breakfast begins, which is followed by a drop in fear, for instance, supports previous suggestions to specifically focus on pre-meal anxiety when treating eating related fears [37, 39, 42]. Further, the findings suggest that body weight exposures using the scale are very suitable to activate various weight related fears that could be targeted during exposure therapy to e.g. increase the tolerance of weighing moments and reduce fears about specific weights on the scale.

Patients with anorexia nervosa were also more anxious, more disgusted and less accepting of virtual bodies with healthier weights than healthy controls. Weight restoration is a key goal of anorexia nervosa recovery [59], but negative emotions associated with healthier body weights such as elevated fear, body disgust or reduced body acceptance, possibly refrain patients from achieving weight restoration and should therefore be targeted during exposure therapy. The patients’ overall emotional reactions after virtual body exposure in the present study, however, were rather low. On the one hand, it could be assumed that our specific VR paradigm was insufficient in eliciting strong emotions, as the participants also had difficulties to imagine that the virtual bodies were theirs. On the other hand, the healthy controls still responded as predicted by reacting less negative with increasing weight of the avatars. Further, the imagination and anxiety levels reported by anorexia nervosa patients in the current study are similar to the results of previous research that even used more personalized avatars, adapted to the participants’ real bodies [60, 61]. This suggests that the low ratings of the patients are not (only) due to limitations of the present VR paradigm, but that other, disorder-specific factors might also play a role.

Contrary to our hypotheses, patients with anorexia nervosa did not prefer the extremely thin virtual bodies over the larger bodies. Rather, they gave undifferentiated and moderately low ratings for all avatars, which may indicate that neither extremely thin nor more healthy-weight virtual bodies were perceived as more acceptable, less disgusting or less frightening by patients. The question arises as to why the reactions of anorexia nervosa patients were less differentiated than those of healthy controls. However, to the authors’ knowledge, no previous research has investigated body acceptance and body disgust during exposure to virtual bodies. Porras-Garcia et al. [60] suggested, for instance, that body image disturbances could account for the low imagination ratings found in their study, and perhaps this also applies to the current findings. Further, disgust, and specifically self-disgust are thought to play an important role in anorexia nervosa (see e.g. [27, 62, 63]. Since the patients had to imagine the virtual bodies were their own bodies, the indifferent disgust and acceptance ratings for all avatars could represent general self-disgust, and perhaps these emotions were evoked by each virtual body, independent of their BMI. Future research is needed to gather more knowledge about what exactly happens when individuals are exposed to bodies in a virtual environment. This is needed to both better understand the processes that underlie the current findings and to derive treatment implications.

After following an intensive exposure intervention next to standard care, patients with anorexia nervosa reported less eating disorder related fears on the questionnaires, were more accepting of healthier weights and consumed more calories despite weight gain; they were less anxious when exposed to food, but their emotional reactions to weight exposure and virtual body exposure only marginally changed. Perhaps, this shows that it is more difficult to treat weight and body related fears with exposure therapy than treating food related fears in anorexia nervosa. It could also mean that our exposure tasks in the laboratory sufficed to activate anorectic fears but were not sensitive enough to capture therapeutic changes, or that insufficient power due to the small sample size exacerbated statistically detecting these changes. Still, the fear reductions we found on the questionnaires assessing eating disorder related fears are comparable or even stronger than the reductions found in previous research [43, 64, 65] and the increase in calorie intake in the present study was larger than in other studies [41, 42]. Nevertheless, the margin of change for some of the measures is small, which is intriguing given the intensity of the treatment that the patients received in the current study. This might, on the one hand, reflect the persistence of anorexia nervosa related fears, the difficulty to achieve fear reductions as well as the need for repeated fear confrontations in order to induce change. On the other hand, the current findings add to prior research by showing the potential and success of exposure therapy in the treatment of anorectic fears (e.g. [26, 37, 39, 41–43, 64, 66].

Our study also has limitations. The sample size of the AN group is small, so replication of the findings is needed. Further, the small sample size does not allow for comparisons between the two anorexia nervosa subtypes. In addition, the recruitment of the healthy control group did not entail diagnostic tools other than assessing current diagnosis, treatment status and EDE-Q scores. By this, it cannot be ruled out that individuals with a potentially undiagnosed mental disorder might be within the sample. The breaks between the weight exposures were short; perhaps, the anxiety levels reported before and after exposure to the actual weight were unduly affected by the preceding exposure to the manipulated higher weight. In the VR paradigm, we only used a specific weight range for the avatars (severely underweight to almost healthy weight); future studies could use a wider range in the avatars BMI and perhaps also include overweight virtual bodies to get a more comprehensive overview of reactions. Further, the order of the three tasks was not randomized, so any confounding effects cannot be detected in this study. Lastly, we could not include a clinical control group that did not receive the exposure treatment. More research is therefore needed to assess the individual effects of the treatments.

### Clinical implications

The results of the food exposure in the laboratory underline that food is a stimulus which activates relevant anorexia nervosa related fears. The outcomes of the exposure treatment confirm that these fears can be effectively treated; so, the use of food exposures to target fears in anorexia nervosa is recommended. The scale also proved a successful modality to trigger anorectic fears. Since emotions related to body and weight seemed more difficult to change in patients with anorexia nervosa using exposure therapy than food related fears, they may require even more intensive exposure treatments than was applied in the current study. Lastly, while this study shows that virtual reality is able to elicit diverse emotional responses in patients with anorexia nervosa that should be targeted during treatment, the outcomes also reveal that much more needs to be learned about when and how virtual reality should be used in interventions to provide the best possible treatment for the patient.

## Conclusion

In conclusion, exposure to food-, body- and weight-related stimuli in the laboratory induces stronger emotional reactions in patients with anorexia nervosa compared to a healthy control group, which is informative for possible exposure therapy targets. It was also found that exposure therapy added to standard care led to reductions in anxiety and increases in calorie consumption and body weight. More research into the cues that elicit fears and targeting of these fears during anorexia nervosa interventions is necessary to develop more effective treatments.

## Data Availability

The datasets used and/or analyzed during the current study are available from the corresponding author on reasonable request.
